# Supplementary material for: Small RNA pathways in the nematode Ascaris in the absence of piRNAs
Source: Nat Commun. 2022 Feb 11;13:837. doi: 10.1038/s41467-022-28482-7 (PMC8837657; doi:10.1038/s41467-022-28482-7)

Supplementary Tables, Figures, and Data Sources

Small RNA Pathways in the Nematode *Ascaris* in the Absence of piRNAs

Maxim V. Zagoskin, Jianbin Wang, Ashley T. Neff, Giovana M. B. Veronezi, and Richard E. Davis

Supplementary Tables

Supplementary Table 1. *Ascaris* small RNA pathway proteins and RNA expression
Supplementary Table 2. *Ascaris* small RNA IP and sequencing libraries

Supplementary Figures

Supplementary Figure 1. AsCSR-1 RNA expression and H3K9me3 marks.
Supplementary Figure 2. *Ascaris* Argonaute antibodies.
Supplementary Figure 3. *Ascaris* male germline regions.
Supplementary Figure 4. *Ascaris* testes 5' all-phosphate vs. 5'-monophosphate small RNAs.
Supplementary Figure 5. Chromosomal location of small RNAs.
Supplementary Figure 6. Distribution and strandedness of small RNAs to mRNAs.
Supplementary Figure 7. Small RNA size distributions in INPUT and AGO IP libraries.
Supplementary Figure 8. Comparisons of siRNA levels and their target RNA expression.
Supplementary Figure 9. Argonaute associated small RNAs targeting Argonaute mRNAs.

Data Source File

Source_Data.xlsx

Supplementary Table 1. *Ascaris* small RNA pathway proteins and RNA expression

Small RNA Pathways	*C. elegans* gene	*Ascaris* chromosome	Start	End	*Ascaris* gene	Strand	GenBank Accession	Description	M1	M2	M3	M4	M5	M6	M7	M8	F1	F2	F3	F4	F5	E1	E2	E3	E4	E5	E6	E7	E8	E9	E10	L1	L2	Muscle	Intestine	Carcass	Highest_expression	Enriched_tissue
**Ascaris Argonautes**	**Catalytic Tetrad**																																					
**AsALG-1**	Yes (D/E/D/H)	chr19	4458043	4471466	ascaris_gene_12048	-	HQ611964	Argonaute ALG-1	22	23	31	28	12	1	1	5	93	54	56	12	3	10	13	18	28	26	39	42	42	58	79	40	8	0	3	2	late_embryo	not_enriched
**AsALG-4**	Yes (D/E/D/D)	chr7	12118054	12141251	ascaris_gene_05665	-	HQ611965	Argonaute ALG-4;	3	2	1	3	34	63	41	4	1	1	1	1	1	2	2	4	3	3	3	3	0	0	1	0	0	0	0	1	testis	testis
**AsALG-5**	Yes (D/E/D/H)	chr18	4714106	4733778	ascaris_gene_11603	-	HQ611966	Argonaute ALG-5	8	5	6	4	2	0	0	1	10	4	5	2	2	3	3	3	6	8	13	7	13	13	8	4	1	1	0	1	late_embryo	not_enriched
**AsALG-6**	NO (D/E/S/H)	chr5	5452999	5472595	ascaris_gene_03788	+	HQ611967	Argonaute ALG-6	24	28	29	21	12	2	1	5	30	16	17	5	5	23	22	26	34	21	25	18	5	2	1	2	0	1	0	1	zygote	not_enriched
**AsALG-7**	Yes (D/E/D/H)	chr11	1414478	1438678	ascaris_gene_07699	-	HQ611968	Argonaute Alg-7	40	44	25	17	20	6	3	2	10	8	8	4	3	9	8	9	15	14	14	10	17	15	10	9	2	2	2	3	testis	testis
**AsWAGO-1**	NO (D/E/D/A)	chr1	15908083	15930379	ascaris_gene_00855	-	HQ611970	Argonaute WAGO-1	81	81	48	92	65	8	4	11	17	12	13	6	10	88	89	80	125	106	75	51	23	10	6	5	1	8	28	65	zygote	not_enriched
**AsWAGO-2**	NO (S/T/G/S)	chr6	12439274	12465887	ascaris_gene_04899	+	HQ611971	Argonaute WAGO-2	25	26	24	24	10	1	1	5	33	31	33	11	10	49	47	42	37	26	31	17	8	6	5	4	1	1	10	4	zygote	zygote
**AsWAGO-3**	Yes (D/E/D/D)	chr18	3708903	3728538	ascaris_gene_11531	+	HQ611972	Argonaute WAGO-3 (C04F12.1-like)	15	16	15	15	9	2	1	3	9	5	5	2	1	2	2	3	3	11	10	10	21	21	19	15	3	3	30	27	intestine	not_enriched
**AsCSR-1**	Yes (D/E/D/D)	chr17	2927127	2946141	ascaris_gene_11056	-	HQ611969	Argonatue CSR-1	109	132	105	122	141	70	35	18	180	96	104	12	29	127	206	241	336	193	131	52	8	3	4	5	1	2	6	3	zygote	zygote
**AsNRDE-3**	NO (D/A/G/R)	chr5	4557517	4582095	ascaris_gene_03731	-	HQ611973	Argonaute NRDE-3 (Nuclear)	19	22	15	13	38	19	6	3	33	19	20	6	6	25	28	34	33	52	50	31	26	24	12	9	2	3	3	3	early_embryo	not_enriched
**Ascaris RdRPs**																																						
**RdRP 1**	ego-1 or rrf-1	chr8	1412361	1451674	ascaris_gene_05790	-	HQ611977	RNA-dependent RNA polymerase RdRP-1	13	14	12	20	37	7	3	2	9	6	6	2	1	4	3	2	4	3	2	1	2	2	3	3	1	0	2	1	testis	testis
**RdRP 2**	rrf-1 or ego-1	chr1	3602126	3640615	ascaris_gene_00191	+	HQ611978	RNA-dependent RNA polymerase RdRP-2	6	6	4	4	3	0	0	1	3	3	2	2	0	1	1	1	3	2	2	2	4	4	4	3	0	0	1	0	low_expression	low_expression
**RdRP 3**	rrf-3	chr2	751145	785712	ascaris_gene_01050	+	HQ611979	RNA-dependent RNA polymerase RdRP-3	11	10	7	16	58	4	2	1	14	7	8	6	2	12	12	15	20	11	11	7	9	6	8	3	1	2	1	1	testis	not_enriched
**miRNA**																																						
	alg-1	chr19	4458043	4471466	ascaris_gene_12048	-	HQ611964	Argonaute ALG-1	22	23	31	28	12	1	1	5	93	54	56	12	3	10	13	18	28	26	39	42	42	58	79	40	8	0	3	2	late_embryo	not_enriched
	dcr-1	chr12	1368927	1402556	ascaris_gene_08345	+	HQ611974	miRNA processing factor	26	26	22	17	15	3	2	2	3	1	1	3	3	2	3	2	5	8	13	11	23	21	22	15	2	7	5	5	late_embryo	not_enriched
	pasha	chr1	1826955	1897512	ascaris_gene_00093	-	HQ611976	miRNA processing factor	43	37	35	26	22	14	7	4	6	4	4	3	1	5	5	6	9	12	10	4	6	5	5	3	0	1	2	1	testis	testis
	drosha	chr15	5528651	5564322	ascaris_gene_10272	+	HQ611975	miRNA processing factor	24	29	26	39	32	4	1	5	13	8	8	4	6	20	16	16	21	41	26	28	41	44	40	28	8	6	3	9	late_embryo	not_enriched
	ain-1	chrX1	1594921	1610151	ascaris_gene_12286	-	JI166032	miRNA RISC complex factor	10	7	8	7	4	1	1	4	67	65	64	10	12	13	15	21	15	22	18	25	29	43	56	50	30	3	6	2	ovary	not_enriched
	nhl-2	chr12	6339984	6370362	ascaris_gene_08672	-	JI165920	Nhl-2	21	22	17	13	12	3	1	3	25	15	16	3	3	13	11	12	11	19	12	11	9	11	11	7	2	1	1	2	early_embryo	not_enriched
	vig-1	chr18	5039655	5051965	ascaris_gene_11629	-		miRNA RISC complex factor, RNA-binding	45	58	53	64	71	23	9	8	32	18	17	7	36	144	199	293	182	236	267	318	191	129	88	51	29	45	41	51	early_embryo	not_enriched
	tsn-1	chrX5	2244380	2260469	ascaris_gene_15329	-		Nuclease domain-containing protein 1	24	28	20	40	35	4	4	21	70	42	43	27	28	36	36	35	36	32	29	21	28	28	42	30	7	114	39	66	muscle	muscle
	cgh-1	chr3	13062450	13096616	ascaris_gene_02547	-		ATP-dependent RNA helicase cgh-1	50	51	46	42	21	4	1	5	42	46	46	12	5	35	27	26	30	50	52	32	22	17	14	11	1	5	3	6	early_embryo	not_enriched
	cgh-1	chr1	96822	134440	ascaris_gene_00004	-		ATP-dependent RNA helicase cgh-1	41	47	40	50	22	3	2	11	62	65	63	20	10	62	54	55	64	76	77	50	33	24	20	13	3	8	6	10	early_embryo	not_enriched
	dcs-1	chr19	1845323	1849979	ascaris_gene_11857	+		Decapping scavenger enzyme, DcpS	4	3	5	9	4	0	0	1	9	4	4	1	1	4	7	6	16	31	20	11	26	20	14	10	1	1	1	1	early_embryo	not_enriched
	xrn-2	chr3	11498424	11507380	ascaris_gene_02441	+		3'-5' exoribonuclease	26	34	24	26	13	1	1	28	35	29	27	14	8	17	15	14	20	17	24	21	21	19	12	8	4	7	13	10	ovary	not_enriched
**ALG-3/4 26G**	missing eri-3 and eri-5																																					
	alg-3/4	chr7	12118054	12141251	ascaris_gene_05665	-	HQ611965	Argonaute ALG-4	3	2	1	3	34	63	41	4	1	1	1	1	1	2	2	4	3	3	3	3	0	0	1	0	0	0	0	1	testis	testis
	rrf-3	chr2	751145	785712	ascaris_gene_01050	+	HQ611979	RNA-dependent RNA polymerase RdRP3	11	10	7	16	58	4	2	1	14	7	8	6	2	12	12	15	20	11	11	7	9	6	8	3	1	2	1	1	testis	not_enriched
	dcr-1	chr12	1368927	1402556	ascaris_gene_08345	+	HQ611974	DICER	26	26	22	17	15	3	2	2	3	1	1	3	3	2	3	2	5	8	13	11	23	21	22	15	2	7	5	5	late_embryo	not_enriched
	eri-1	chr5	8275856	8320784	ascaris_gene_03925	-	JI173279	ERI1 exoribonuclease 3	15	13	7	11	16	8	6	13	29	23	20	5	6	26	26	27	21	24	23	25	17	15	24	27	11	6	26	11	intestine	not_enriched
	rde-4	chr3	5134164	5146005	ascaris_gene_02096	-	JI170472	dsRNA binding domain-containing protein rde-4	14	17	19	20	11	2	2	2	11	5	6	2	2	7	9	11	11	15	15	16	19	16	13	9	5	3	11	4	late_embryo	not_enriched
	drh-3	chr9	10645994	10673120	ascaris_gene_06989	-	JI165061	ATP-dependent RNA helicase	41	47	41	71	70	10	5	10	15	13	13	14	11	28	23	16	20	17	14	19	21	24	16	14	8	6	11	13	testis	testis
	gstf-1	chr13	4404093	4411376	ascaris_gene_09121	-		Gametocyte-specific factor 1-like (GSTF-1)	13	15	10	12	7	2	1	1	4	3	2	2	4	14	10	8	12	13	10	8	17	19	14	12	1	2	1	3	late_embryo	late_embryo
	pir-1	chr17	1204210	1221260	ascaris_gene_10954	-	JI172438	R/RNP complex-1-interacting phosphatase	75	74	70	77	89	12	24	9	22	19	21	28	71	150	83	59	67	75	76	54	78	66	62	34	16	22	34	40	zygote	not_enriched
**CSR-1 22G**																																						
	csr-1	chr17	2927127	2946141	ascaris_gene_11056	-	HQ611969	Argonatue CSR-1	109	132	105	122	141	70	35	18	180	96	104	12	29	127	206	241	336	193	131	52	8	3	4	5	1	2	6	3	zygote	zygote
	ego-1	chr8	1412361	1451674	ascaris_gene_05790	-	HQ611977	RNA-dependent RNA polymerase	13	14	12	20	37	7	3	2	9	6	6	2	1	4	3	2	4	3	2	1	2	2	3	3	1	0	2	1	testis	testis
	wago-2	chr6	12439274	12465887	ascaris_gene_04899	+	HQ611971	Argonaute WAGO-2	25	26	24	24	10	1	1	5	33	31	33	11	10	49	47	42	37	26	31	17	8	6	5	4	1	1	10	4	zygote	zygote
	ekl-1	chr19	1737257	1748267	ascaris_gene_11849	-		Tudor domain containing protein	11	11	14	15	13	3	1	2	10	6	7	3	3	15	10	9	15	13	10	5	16	18	12	7	1	1	1	1	late_embryo	not_enriched
	drh-3	chr9	10645994	10673120	ascaris_gene_06989	-	JI165061	ATP-dependent RNA helicase	41	47	41	71	70	10	5	10	15	13	13	14	11	28	23	16	20	17	14	19	21	24	16	14	8	6	11	13	testis	testis
	cde-1	chr14	2083211	2109048	ascaris_gene_09522	+	JI167978	Terminal uridylyltransferase 7 (cde-1, cid-1, pup-1)	13	14	18	17	17	24	20	2	5	3	3	3	2	4	5	6	7	10	11	10	19	22	22	15	2	0	2	1	late_embryo	not_enriched
	pir-1	chr17	1204210	1221260	ascaris_gene_10954	-	JI172438	R/RNP complex-1-interacting phosphatase	75	74	70	77	89	12	24	9	22	19	21	28	71	150	83	59	67	75	76	54	78	66	62	34	16	22	34	40	zygote	not_enriched
	pir-1	chr17	1204210	1221260	ascaris_gene_10954	-	JI172438	R/RNP complex-1-interacting phosphatase	75	74	70	77	89	12	24	9	22	19	21	28	71	150	83	59	67	75	76	54	78	66	62	34	16	22	34	40	zygote	not_enriched
**WAGO 22G (Mutator)**	missing rsd-6, mut-2, mut-8, mut-15, mut-16, rde-10, and rde-11																																					
	wago-1	chr1	15908083	15930379	ascaris_gene_00855	-	HQ611970	Argonaute WAGO-1	81	81	48	92	65	8	4	11	17	12	13	6	10	88	89	80	125	106	75	51	23	10	6	5	1	8	28	65	zygote	not_enriched
	wago-2	chr6	12439274	12465887	ascaris_gene_04899	+	HQ611971	Argonaute WAGO-2	25	26	24	24	10	1	1	5	33	31	33	11	10	49	47	42	37	26	31	17	8	6	5	4	1	1	10	4	zygote	zygote
	wago-2	chr6	12439274	12465887	ascaris_gene_04899	+	HQ611971	Argonaute WAGO-2	25	26	24	24	10	1	1	5	33	31	33	11	10	49	47	42	37	26	31	17	8	6	5	4	1	1	10	4	zygote	zygote
	nrde-3	chr5	4557517	4582095	ascaris_gene_03731	-	HQ611973	Argonaute NRDE-3 (nuclear)	19	22	15	13	38	19	6	3	33	19	20	6	6	25	28	34	33	52	50	31	26	24	12	9	2	3	3	3	early_embryo	not_enriched
	rrf-1	chr1	3602126	3640615	ascaris_gene_00191	+	HQ611978	RNA-dependent RNA polymerase RdRP1	6	6	4	4	3	0	0	1	3	3	2	2	0	1	1	1	3	2	2	2	4	4	4	3	0	0	1	0	low_expression	low_expression
	drh-3	chr9	10645994	10673120	ascaris_gene_06989	-	JI165061	ATP-dependent RNA helicase	41	47	41	71	70	10	5	10	15	13	13	14	11	28	23	16	20	17	14	19	21	24	16	14	8	6	11	13	testis	testis
	mut-7	chr13	5535757	5559199	ascaris_gene_09190	+	JI166580	putative exonuclease mut-7	35	42	33	34	13	2	1	3	4	2	2	5	5	19	18	16	21	25	33	18	20	20	15	10	1	6	6	6	early_embryo	not_enriched
	mut-14	chr7	10375260	10397419	ascaris_gene_05550	+		ATP-dependent RNA helicase	8	9	8	12	16	3	1	2	4	3	3	2	2	7	6	8	7	12	11	11	17	20	21	16	2	1	2	2	late_embryo	late_embryo
	pir-1	chr17	1204210	1221260	ascaris_gene_10954	-	JI172438	R/RNP complex-1-interacting phosphatase	75	74	70	77	89	12	24	9	22	19	21	28	71	150	83	59	67	75	76	54	78	66	62	34	16	22	34	40	zygote	not_enriched
	pir-1	chr17	1204210	1221260	ascaris_gene_10954	-	JI172438	R/RNP complex-1-interacting phosphatase	75	74	70	77	89	12	24	9	22	19	21	28	71	150	83	59	67	75	76	54	78	66	62	34	16	22	34	40	zygote	not_enriched
	rde-12	chr11	748938	787211	ascaris_gene_07665	+		ATP-dependent RNA helicase	37	30	33	35	39	6	5	3	23	24	26	13	44	82	72	104	101	102	82	48	41	29	20	23	6	10	81	13	zygote	not_enriched
	rsd-3	chrX1	11456622	11476214	ascaris_gene_12861	+		Required for RNAi spreading	10	11	10	17	18	2	1	4	29	23	22	7	4	5	4	3	5	11	8	6	10	14	17	10	4	4	2	4	ovary	not_enriched
**Nuclear or heritable RNAi**	missing hrde-1, nrde-1, and nrde-4																																					
	nrde-3	chr5	4557517	4582095	ascaris_gene_03731	-	HQ611973	Argonaute NRDE-3 (nuclear)	19	22	15	13	38	19	6	3	33	19	20	6	6	25	28	34	33	52	50	31	26	24	12	9	2	3	3	3	early_embryo	not_enriched
	nrde-2	chr17	2809330	2839907	ascaris_gene_11047	-	JI165799	NRDE-2 homolog	27	29	27	29	27	15	6	3	6	3	4	2	3	7	9	14	10	12	13	14	15	16	18	13	4	2	3	3	testis	not_enriched
	ZNFX-1 (hrr-1)	chr8	10979423	11018480	ascaris_gene_06342	-		nfx1-type zinc finger-containing protein 1	20	20	16	14	8	1	1	2	13	19	19	8	6	17	24	31	43	44	58	60	19	6	4	4	0	1	9	4	early_embryo	early_embryo
**Exogenous RNAi**	missing rde-1, rde-8, sid-2, nyn-1, and nyn-2																																					
	dcr-1	chr12	1368927	1402556	ascaris_gene_08345	+	HQ611974	DICER - small RNA processing factor	26	26	22	17	15	3	2	2	3	1	1	3	3	2	3	2	5	8	13	11	23	21	22	15	2	7	5	5	late_embryo	not_enriched
	rde-4	chr3	5134164	5146005	ascaris_gene_02096	-	JI170472	dsRNA binding domain-containing protein	14	17	19	20	11	2	2	2	11	5	6	2	2	7	9	11	11	15	15	16	19	16	13	9	5	3	11	4	late_embryo	not_enriched
	drh-3	chr9	10645994	10673120	ascaris_gene_06989	-	JI165061	ATP-dependent RNA helicase	41	47	41	71	70	10	5	10	15	13	13	14	11	28	23	16	20	17	14	19	21	24	16	14	8	6	11	13	testis	testis
	drh-1	chr5	11170692	11199110	ascaris_gene_04085	-	JI168098	ATP-dependent RNA helicase	7	8	6	11	49	21	6	1	3	1	1	2	1	2	3	3	6	6	7	6	28	26	23	24	3	2	5	3	late_embryo	late_embryo
	rrf-1	chr1	3602126	3640615	ascaris_gene_00191	+	HQ611978	RNA-dependent RNA polymerase RdRP1	6	6	4	4	3	0	0	1	3	3	2	2	0	1	1	1	3	2	2	2	4	4	4	3	0	0	1	0	low_expression	low_expression
	sid-1	chr11	5446384	5466010	ascaris_gene_07963	+	JI169268	Systemic RNAi (RNA transporter)(RNAi spreading)	28	30	27	25	41	11	8	3	27	16	17	6	6	10	11	12	14	16	16	16	26	26	23	18	5	1	17	2	late_embryo	not_enriched
**piRNA (21U) - absent**	missing prg-1 and henn-1																																					
**ERGO-1 26G - absent**	missing ergo-1, henn-1, eri-9, mut-16, eri-6, eri-7, eri-3, and eri-5																																					
	eri-1	chr16	4855339	4866790	ascaris_gene_10712	-	JI173279	3'-5' exonuclease eri-1	8	9	7	7	6	1	1	1	3	2	2	1	3	6	6	7	9	5	4	4	3	3	2	1	1	0	0	1	zygote	not_enriched
	drh-3	chr9	10645994	10673120	ascaris_gene_06989	-	JI165061	putative atp-dependent r helicase ddx58	41	47	41	71	70	10	5	10	15	13	13	14	11	28	23	16	20	17	14	19	21	24	16	14	8	6	11	13	testis	testis
	rde-4	chr3	5134164	5146005	ascaris_gene_02096	-	JI170472	dsRNA binding domain-containing protein rde-4	14	17	19	20	11	2	2	2	11	5	6	2	2	7	9	11	11	15	15	16	19	16	13	9	5	3	11	4	late_embryo	not_enriched
**Other genes**	missing deps-1, meg-3, meg-4 , rsd-2, sid-2, simr-1, smut-1																																					
	emb-4	chr11	1732106	1763591	ascaris_gene_07721	+		intron-binding protein aquarius (EMB-4 helicase)	14	19	16	16	7	1	1	5	7	5	5	2	1	3	3	3	6	9	10	9	9	8	6	5	2	1	1	1	testis	not_enriched
	morc-1	chr4	12620863	12650816	ascaris_gene_03369	+		MORC family CW-type zinc finger protein 2A	22	24	19	19	9	1	1	4	14	11	11	7	6	21	12	8	13	22	21	15	31	28	18	20	2	4	3	11	late_embryo	not_enriched
	smg-2	chrX1	21840121	21870448	ascaris_gene_13435	-	JI164253	Regulator of nonsense transcripts 1 (smg-2)	34	41	39	45	30	3	1	11	62	27	28	7	12	49	59	77	63	94	63	63	77	92	62	26	8	4	9	7	late_embryo	not_enriched

Supplementary Table 2. *Ascaris* small RNA IP and sequencing libraries

Library	INPUT/AGO	Developmental stage	Reads # (18-30nt)	Norm (30 M)
ALG1_testis	ALG1	testis	20,258,575	1.48
ALG1_ovary	ALG1	ovary	25,894,531	1.16
ALG1_4cell	ALG1	4cell	6,041,327	4.97
ALG4_M1	ALG4	M1	21,920,380	1.37
ALG4_M2	ALG4	M2	17,303,681	1.73
ALG4_M3	ALG4	M3	20,147,308	1.49
ALG4_M4	ALG4	M4	17,019,710	1.76
ALG4_M5	ALG4	M5	14,594,971	2.06
ALG4_M6	ALG4	M6	33,061,039	0.91
ALG4_M7	ALG4	M7	20,076,957	1.49
ALG4_testis	ALG4	testis	24,413,409	1.23
ALG4_ovary	ALG4	ovary	13,781,368	2.18
ALG4_4cell	ALG4	4cell	11,785,658	2.55
WAGO1_M1	WAGO1	M1	64,894,596	0.46
WAGO1_M2	WAGO1	M2	27,650,140	1.08
WAGO1_M3	WAGO1	M3	78,808,041	0.38
WAGO1_M4	WAGO1	M4	51,542,968	0.58
WAGO1_M5	WAGO1	M5	46,436,116	0.65
WAGO1_M6	WAGO1	M6	34,158,119	0.88
WAGO1_M7	WAGO1	M7	35,436,541	0.85
WAGO1_testis	WAGO1	testis	116,225,461	0.26
WAGO1_ovary	WAGO1	ovary	81,649,947	0.37
WAGO1_4cell	WAGO1	4cell	45,550,387	0.66
WAGO2_M1	WAGO2	M1	19,368,275	1.55
WAGO2_M2	WAGO2	M2	12,643,822	2.37
WAGO2_M3	WAGO2	M3	17,922,560	1.67
WAGO2_M4	WAGO2	M4	12,396,772	2.42
WAGO2_M5	WAGO2	M5	21,504,190	1.40
WAGO2_M6	WAGO2	M6	41,636,168	0.72
WAGO2_M7	WAGO2	M7	29,775,560	1.01
WAGO2_testis	WAGO2	testis	41,814,032	0.72
WAGO2_ovary	WAGO2	ovary	32,028,287	0.94
WAGO2_4cell	WAGO2	4cell	27,291,379	1.10
CSR1_M1	CSR1	M1	49,341,217	0.61
CSR1_M2	CSR1	M2	10,181,731	2.95
CSR1_M3	CSR1	M3	56,729,787	0.53
CSR1_M4	CSR1	M4	45,346,435	0.66
CSR1_M5	CSR1	M5	63,015,414	0.48
CSR1_M6	CSR1	M6	6,587,238	4.55
CSR1_M7	CSR1	M7	20,430,927	1.47
CSR1_testis	CSR1	testis	84,083,235	0.36
CSR1_ovary	CSR1	ovary	36,661,269	0.82
CSR1_4cell	CSR1	4cell	86,131,360	0.35
WAGO3_M1	WAGO3	M1	41,338,185	0.73
WAGO3_M2	WAGO3	M2	13,452,144	2.23
WAGO3_M3	WAGO3	M3	23,253,295	1.29
WAGO3_M4	WAGO3	M4	16,275,541	1.84
WAGO3_M5	WAGO3	M5	44,131,606	0.68
WAGO3_M6	WAGO3	M6	20,300,969	1.48
WAGO3_M7	WAGO3	M7	20,213,402	1.48
WAGO3_testis	WAGO3	testis	29,132,141	1.03
WAGO3_ovary	WAGO3	ovary	22,265,707	1.35
WAGO3_4cell	WAGO3	4cell	40,916,382	0.73
NRDE3_M1	NRDE3	M1	24,032,409	1.25
NRDE3_M2	NRDE3	M2	28,945,864	1.04
NRDE3_M3	NRDE3	M3	15,718,655	1.91
NRDE3_M4	NRDE3	M4	6,300,983	4.76
NRDE3_M5	NRDE3	M5	21,045,060	1.43
NRDE3_M6	NRDE3	M6	30,055,113	1.00
NRDE3_M7	NRDE3	M7	17,823,901	1.68
NRDE3_testis	NRDE3	testis	70,567,291	0.43
NRDE3_ovary	NRDE3	ovary	62,473,304	0.48
NRDE3_4cell	NRDE3	4cell	17,267,975	1.74

Supplementary Figure legends:

Supplementary Figure 1. AsCSR-1 RNA expression and H3K9me3 marks. A genome browser view on Chr17 shows the AsCSR-1 gene (in between 2.9 - 2.95 Mb, marked by an arrow) and other gene models in this region of the genome, RNA-seq and H3K9me3 ChIP-seq from selected developmental stages. The gene models are largely based on RNA-seq (including ISO-seq) data. Red and blue indicate forward and reverse transcription, respectively. Note the high expression of AsCSR-1 in early embryos (1-4 cells) and the loss of its expression in 32-64 cells associated with heavy H3K9me3 marks.

Supplementary Figure 2. *Ascaris* Argonaute antibodies. A. Western blots illustrating the specificity of antibodies and the relative amount of Argonaute proteins in the total cell lysate (T), cytoplasmic (C), or nuclear (N) fraction. The size of the Argonaute proteins is at ~ 100 kDa. An H3 histone variant (CENP-A) is used as a control for the cell fractionation. Data are derived from two or more biological replicates. **B.** Argonautes IP small RNAs and Northern blots. Illustrated are small RNAs that co-IP with selected Argonautes antibodies on gels or from Northern blots. Note the high specificity of AsALG-1 IP for miRNAs and AsWAGO-1 for 22G-RNAs. CBP80 is a negative control that does not bind to any small RNAs. Data are derived from two biological replicates. miR-100 sequence: AACCCGTAGATCCGAACTTGTGTT; Northern probe: CACAAGTTCGGATCTACGG. 22G-49 sequence: GTTAACGTAGAGCTCGCCAGAG; Northern probe: TGGCGAGCTCTACGTTAAC.

Supplementary Figure 3. *Ascaris* male germline regions. Male germline removed and untangled in an 11"x10'' tray in 1x PBS. The germinal tip is less than 0.15 mm in diameter and typically entangled around a downstream region of the germline; thus it requires careful dissection to obtain the intact region.

Supplementary Figure 4. *Ascaris* testes 5' all-phosphate vs. 5'-monophosphate small RNA libraries. Comparison of small RNA library preparation methods (see methods) that capture 5' all-phosphate vs. 5'-monophosphate in broad regions of the testis. Region 1 corresponds to the mitotic region, transition zone, and early pachytene, Region 2 to late pachytene and meiosis, and Region 3 to late meiosis and mature spermatids. We observed an increased amount of small RNAs matching rRNAs in region 3 that have a random size distribution, consistent with what is observed in spermatids (Fig. 4C). This may be associated with the turnover of rRNAs in *Ascaris* late meiosis. Note the enrichment of 5'-monophosphate small RNAs on right, i.e. miRNAs in Region 1 and 26G-RNAs in Region 2.

Supplementary Figure 5. Chromosomal location of small RNAs. Distribution of small RNAs associated with A) Ascaris CSR-1 (green) and B) WAGO-1 (Red) Argonautes on *Ascaris* chromosomes. The small RNAs are normalized to the total number of sequencing reads. The scale on Y-axis are (provide scale of 0- X reads) capped so plots at highly enriched regions will not go beyond the top lines. Note the relative even distribution of AsCSR-1 associated small RNAs (targeting mRNAs) and the more biased distribution of AsWAGO-1 associated small RNAs (targeting repeats) in the genomic regions that will be eliminated during programmed DNA elimination. These eliminated regions have higher levels of repetitive sequences. Also note that the sex chromosomes are largely silenced in the male germline, thus there is a lack of siRNAs associated with AsCSR-1 on these chromosomes in the testis.

Supplementary Figure 6. Distribution and strandedness of small RNAs to mRNAs. Meta-analysis of siRNA distribution across mRNAs (in 100 bins on x-axis). The relative levels of the siRNAs are shown on y-axis. Note the biased antisense targeting at the 5'-end of mRNAs at late pachytene and meiosis for many of the Argonaute associated small RNAs, suggesting a possible concerted effort to repress the mRNAs for their clearance. Blue represents antisense to mRNAs and red indicates sense mRNAs.

Supplementary Figure 7. Small RNA size distributions in INPUT and AGO IP libraries. The size distribution and frequency of small RNAs associated with the INPUT or a specific Argonaute IP. Small RNAs (18-30 nt) from input and Argonaute IP were plotted for the whole sequencing library (All-siRNAs) and different types of small RNA targets. Small RNAs starting with A, C, G, and U of different sizes (x-axis)

were plotted against their read frequencies (y-axis; raw reads in millions). For each library, the y-axis scale is set the same as All-siRNAs to provide a direct comparison between different types of siRNAs within the same library.

Supplementary Figure 8. Comparisons of siRNA levels and their target RNA expression. Pairwise comparisons of siRNAs associated with different argonautes and their targets. **A.** Repetitive sequences targeted by small RNAs in the testis (data from Figure 2A). The small RNAs are normalized to the total number of sequencing reads (see methods). The RNA level (in rpkm) for these repeat targets on the y-axis is derived from testis RNA-seq data. Overall, the RNA expression level from these repeats is low, suggesting a potential repression role of the siRNAs. Note the similarity between AsWAGO-1, AsWAGO-2 and AsNRDE-3. **B.** mRNA small RNA targets from the testis in late pachytene and meiosis (M6). The red indicates mRNAs that are specifically expressed during male meiosis. Note the differential targeting between AsCSR-1 and AsALG-4 and the overlap between AsNRDE-3 and AsCSR-1. The targeted mRNAs show complex RNA expression levels, suggesting a potential mix of licensing, tuning and repressive functions for the small RNAs.

Supplementary Figure 9. Argonaute associated small RNAs targeting Argonaute mRNAs. Genome browser tracks showing four Argonautes mRNAs targeted by their siRNAs in different developmental stages. The mRNA levels are normalized RNA-seq data from the M1-M8, ovary, and 4-cell embryos. PRO-seq data shows the nascent RNAs for these four Argonaute genes. The data below the PRO-seq are small RNA sequencing data from the corresponding Argonaute IPs. This illustrates potential auto-regulation of the expression of Argonautes by their associated small RNAs. For example, AsALG-4 is meiosis-specific and its expression in M5-M7 leads to the generation of 26G-RNAs that also target and repress AsALG-4 mRNA in M6-M7.

Supplementary Figure 1

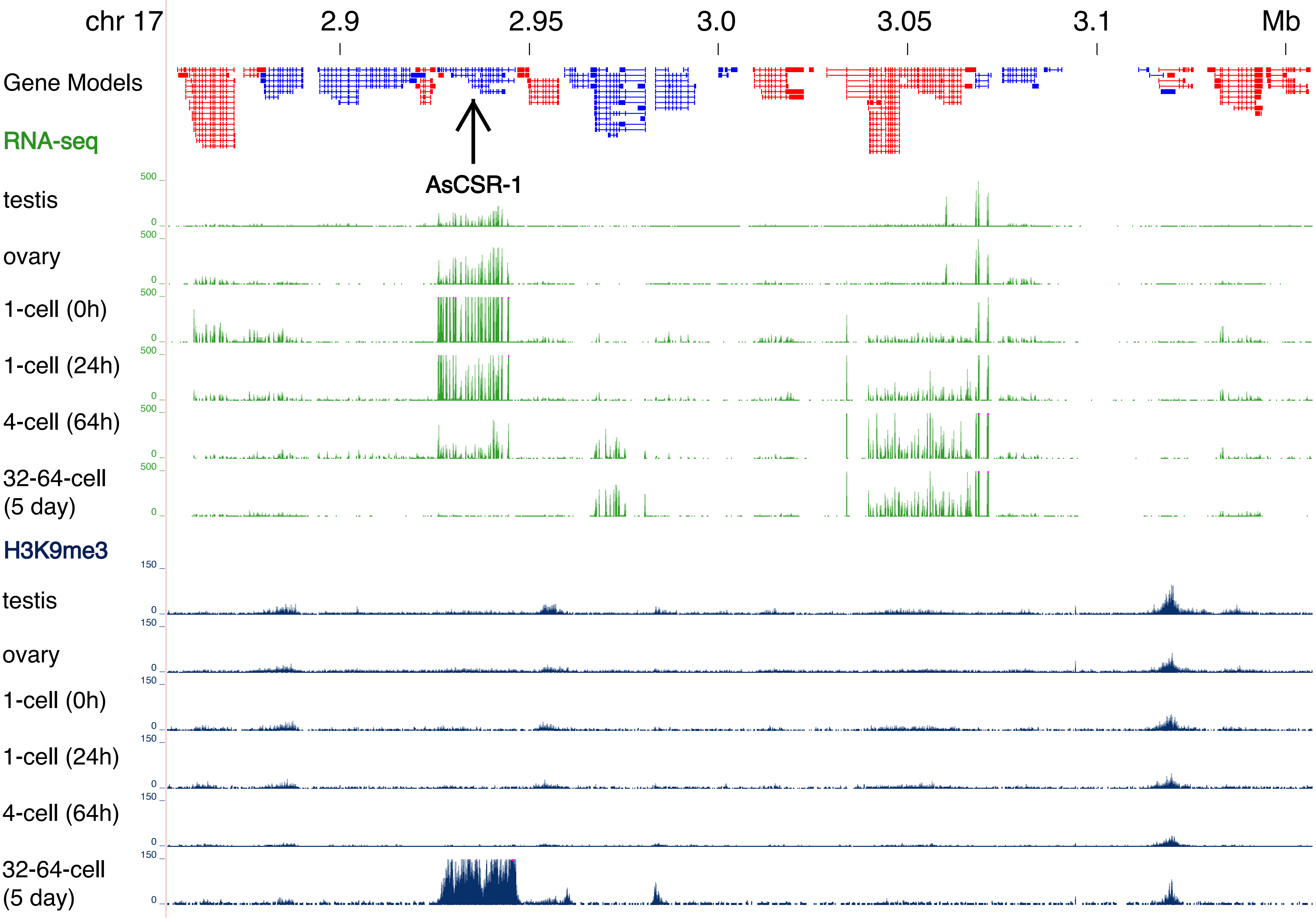

Supplementary Figure 2

A

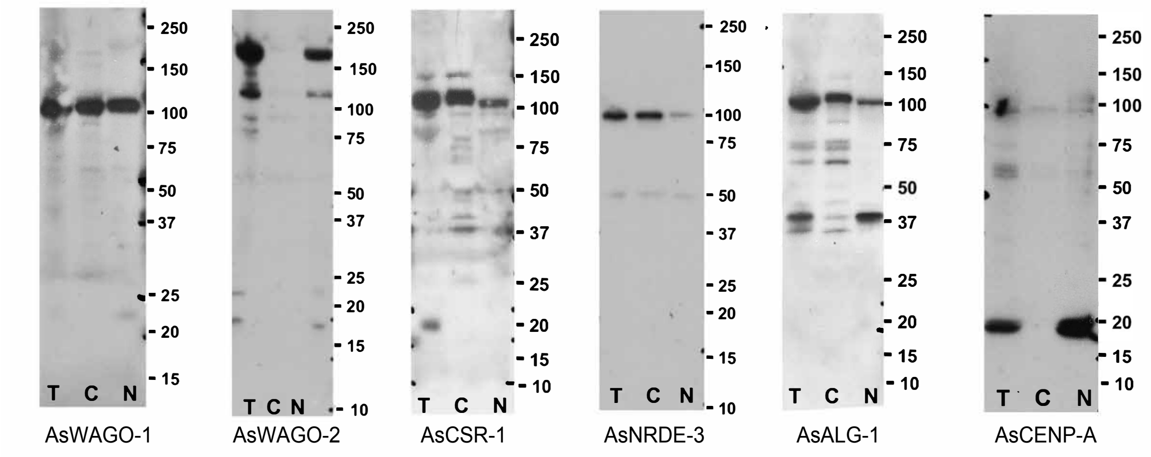

B

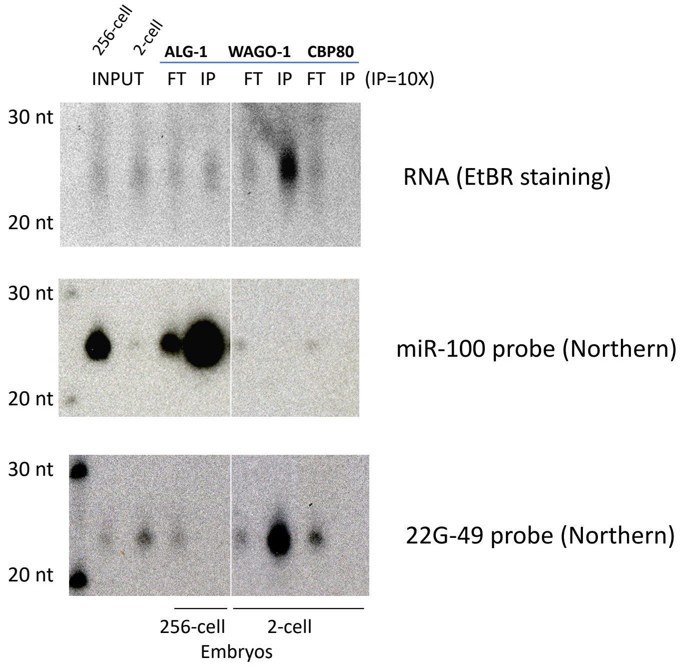

Supplementary Figure 3

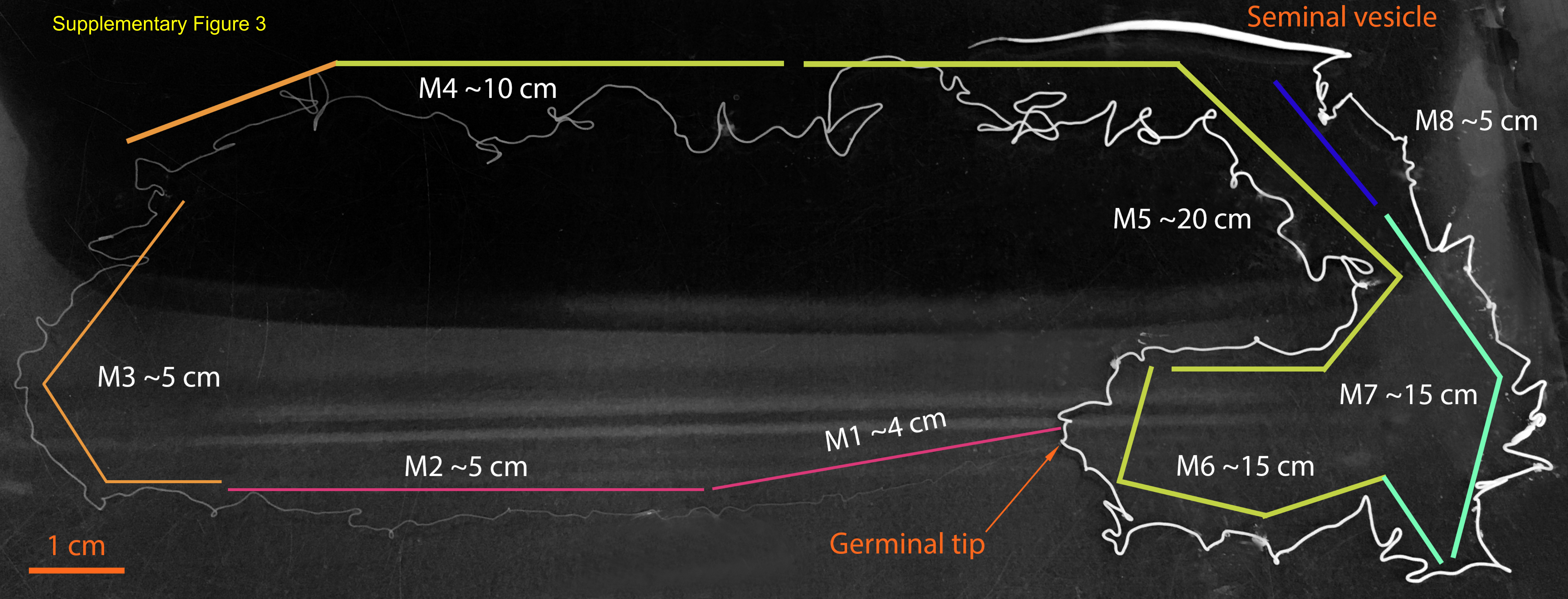

Supplementary Figure 4

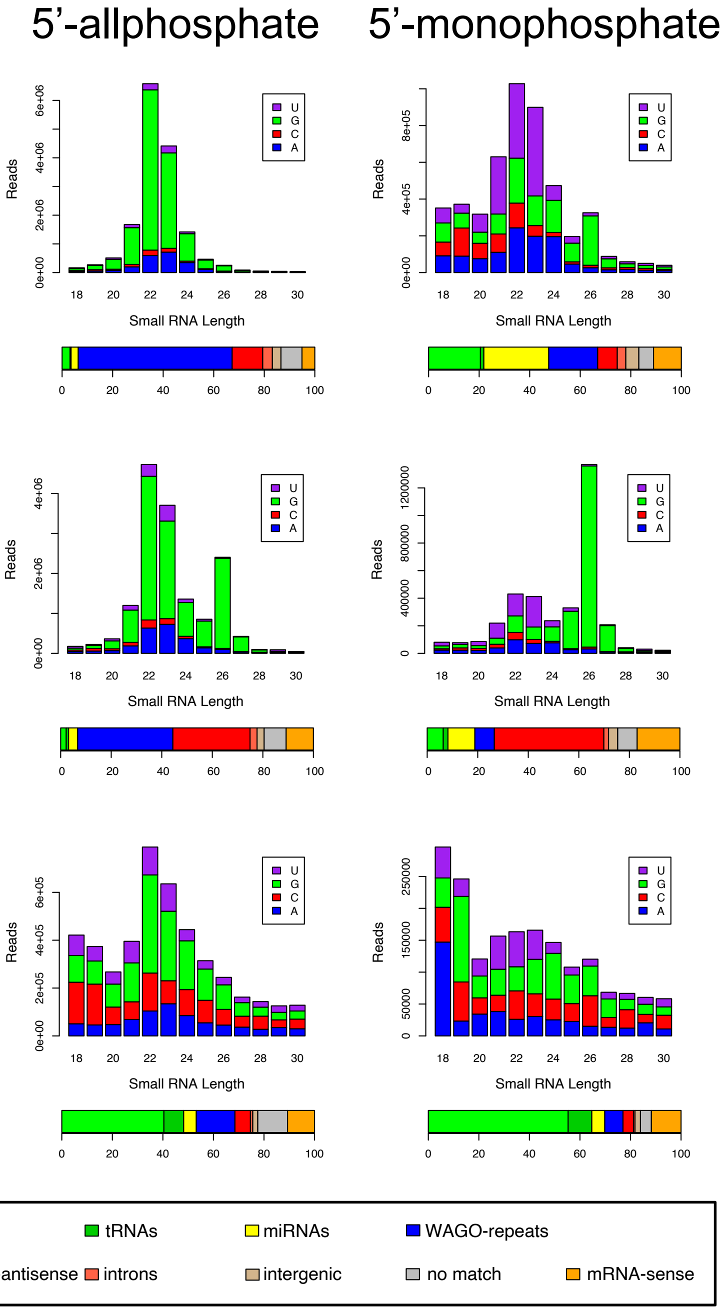

Supplementary Figure 5a

siRNAs associated with CSR-1 (green) and WAGO-1 (red) on *Ascaris* Chromosomes in Testis

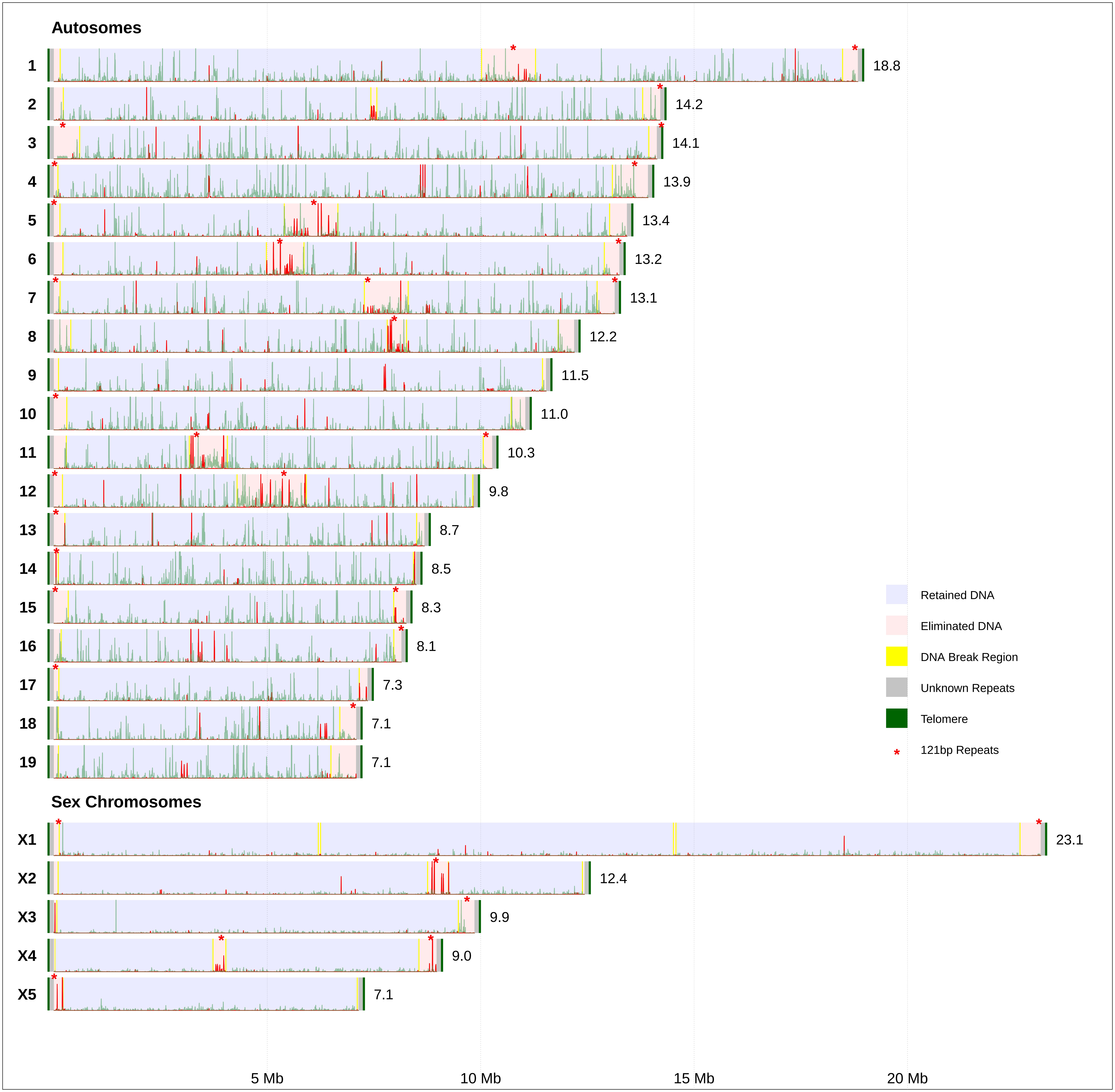

Supplementary Figure 5b

siRNAs associated with CSR-1 (green) and WAGO-1 (red) on *Ascaris* Chromosomes in Ovary

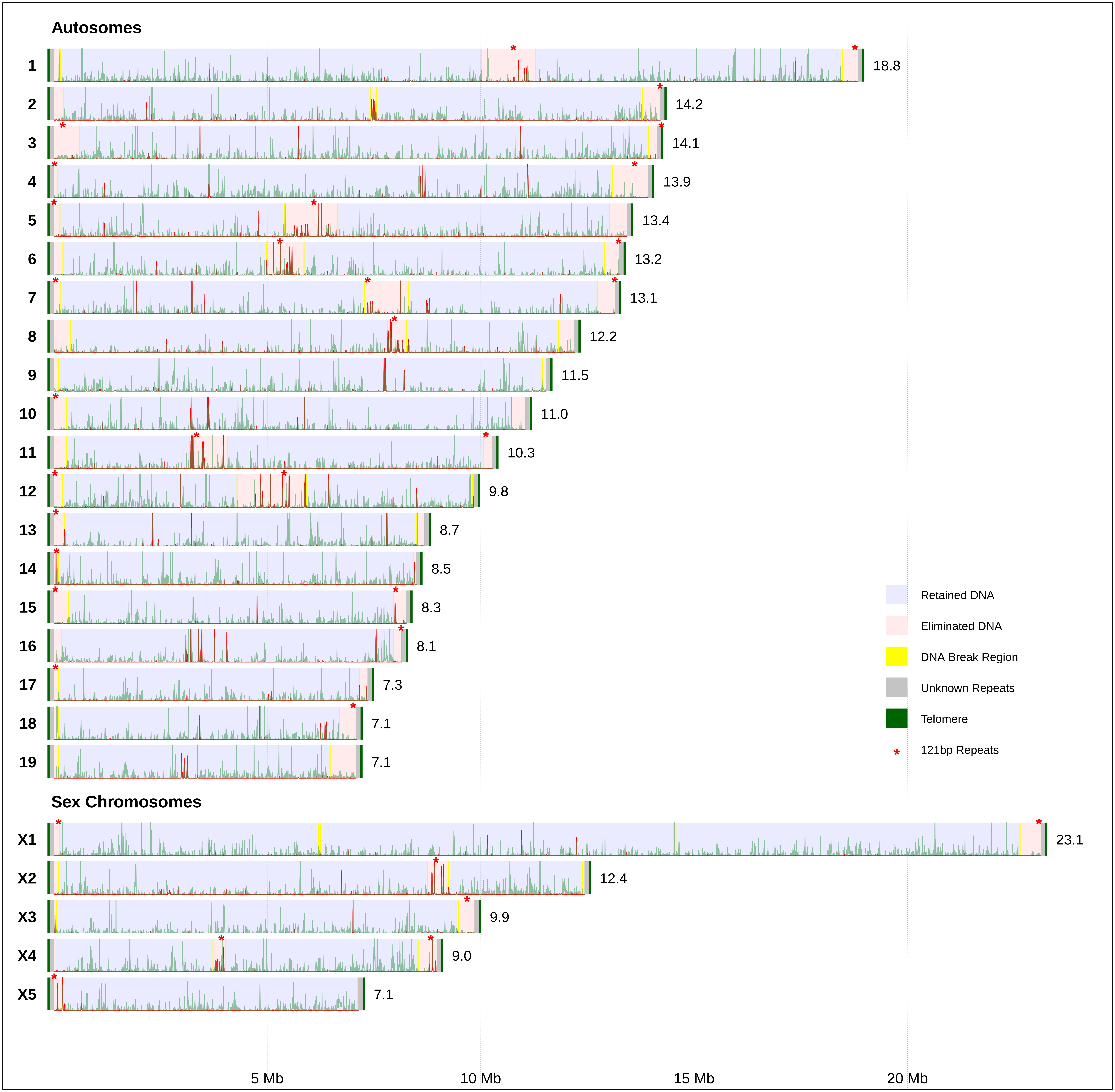

Supplementary Figure 6

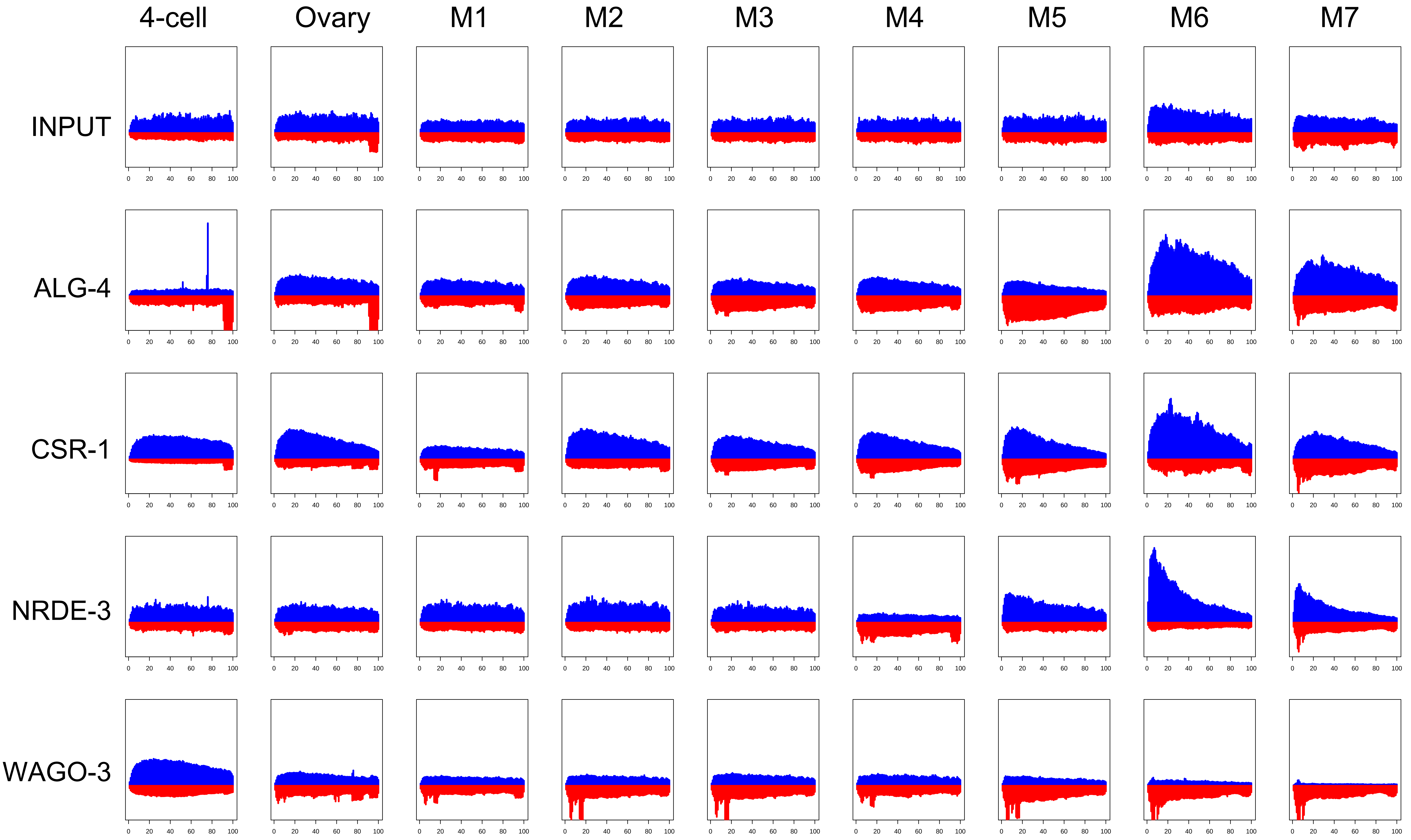

Supplementary Figure 7a

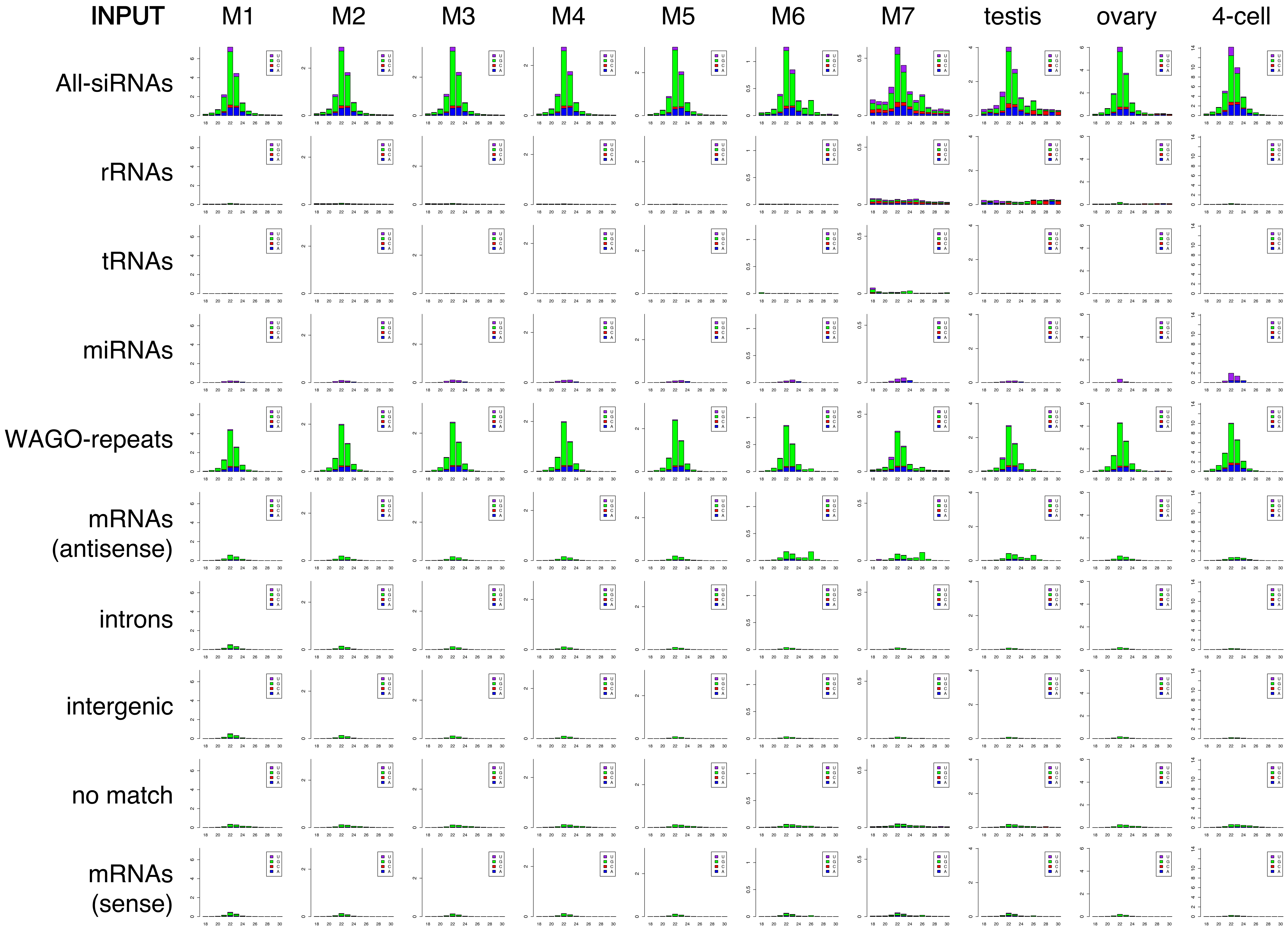

Supplementary Figure 7b

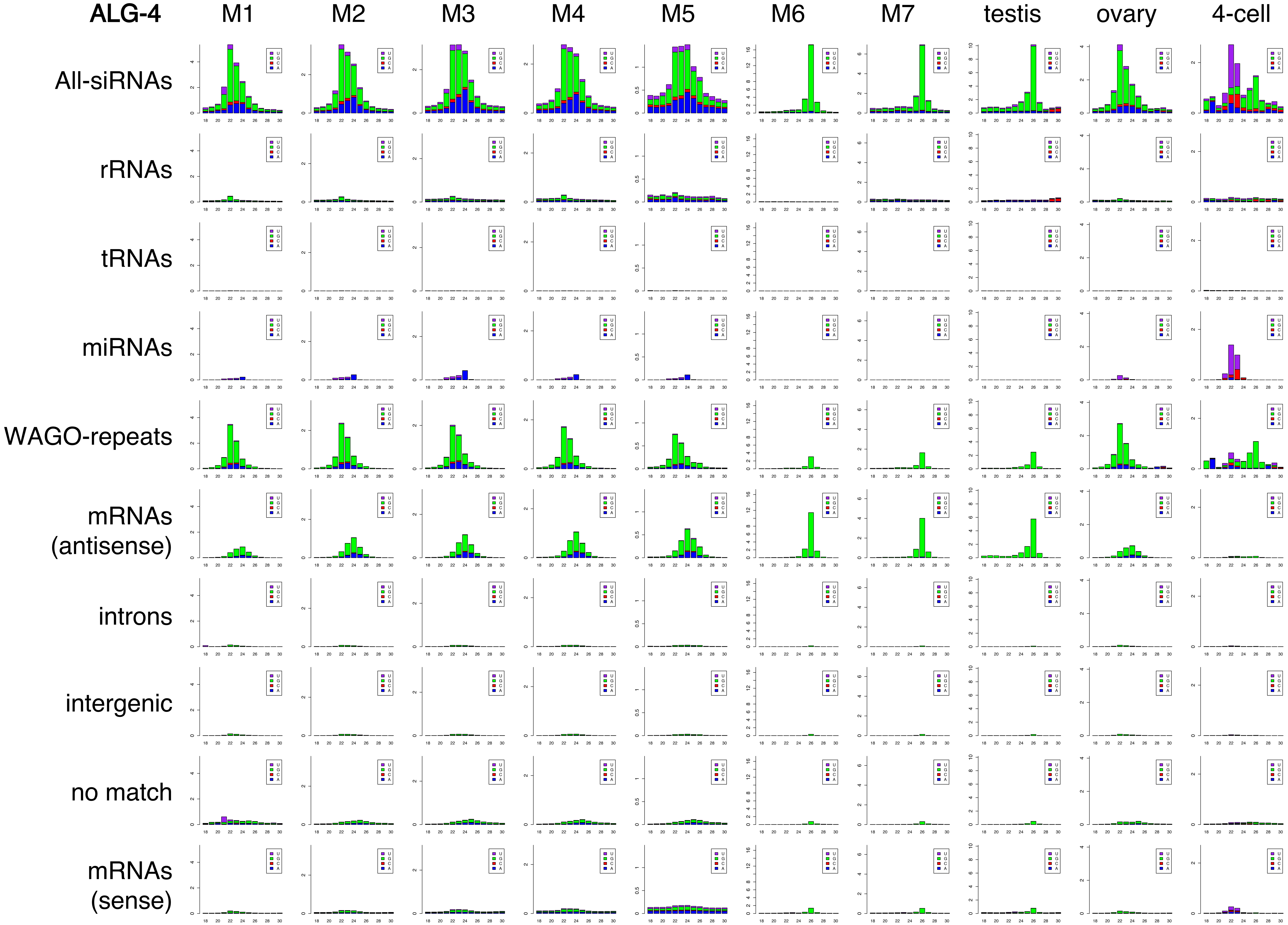

Supplementary Figure 7c

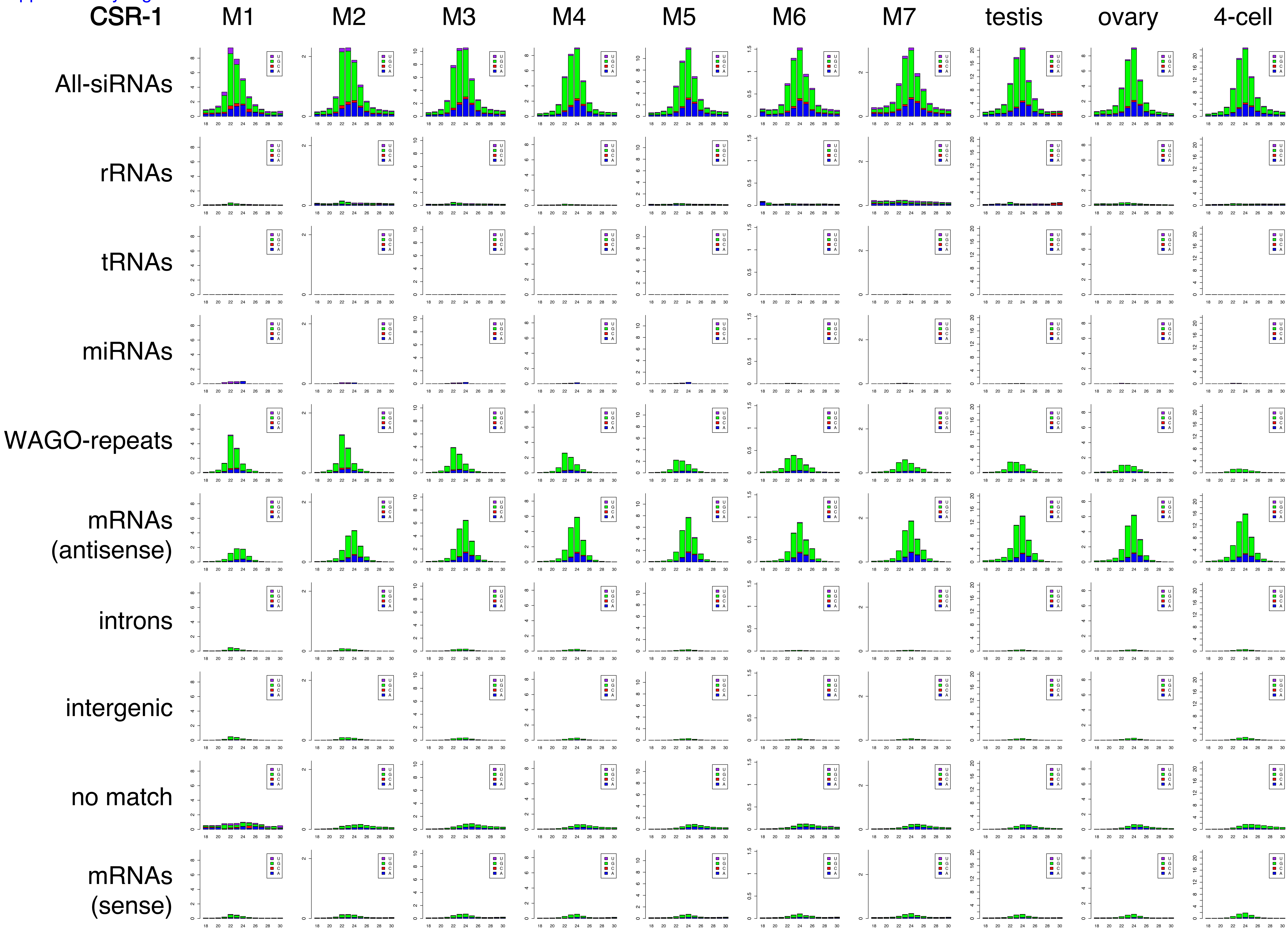

Supplementary Figure 7d

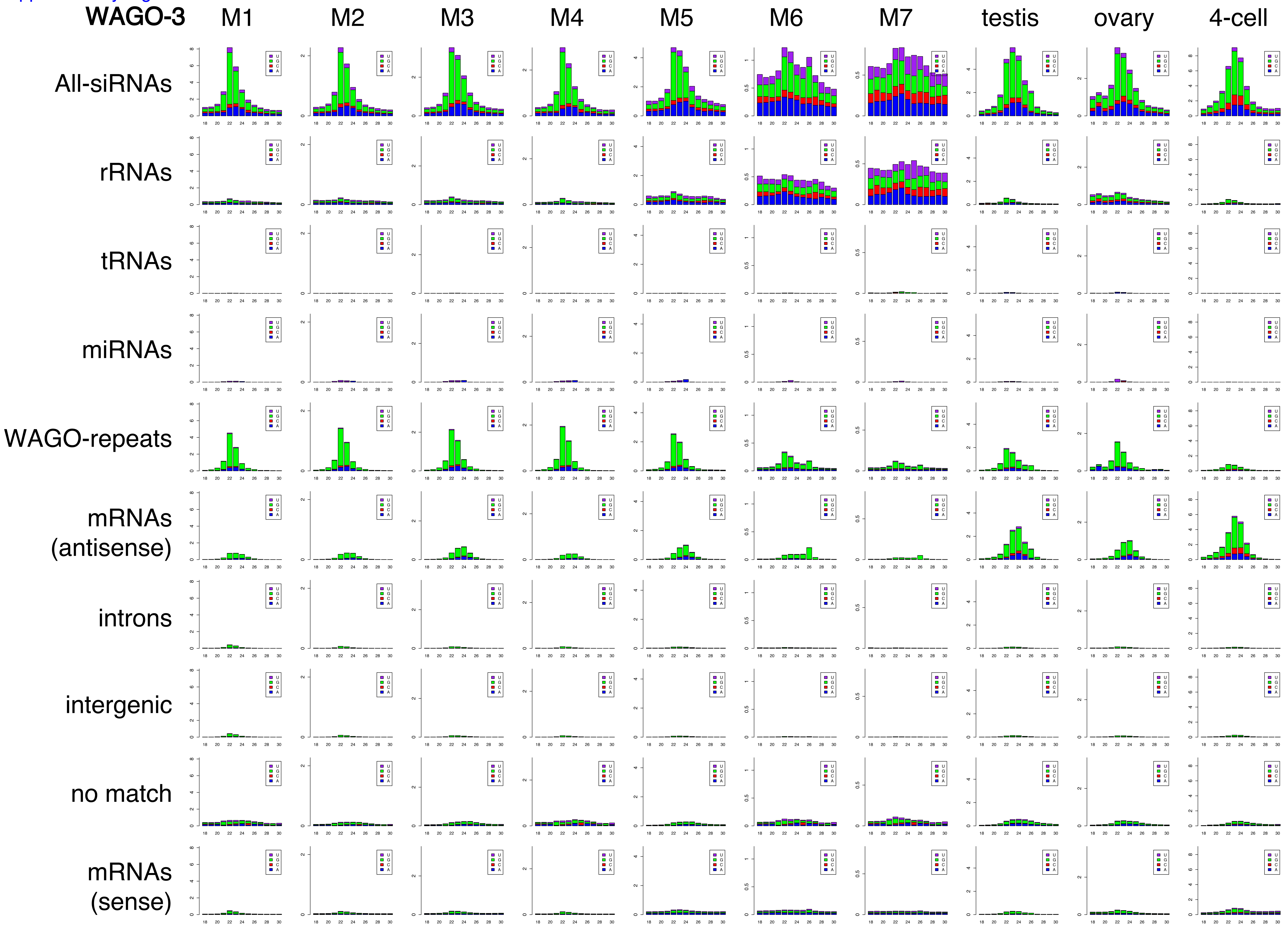

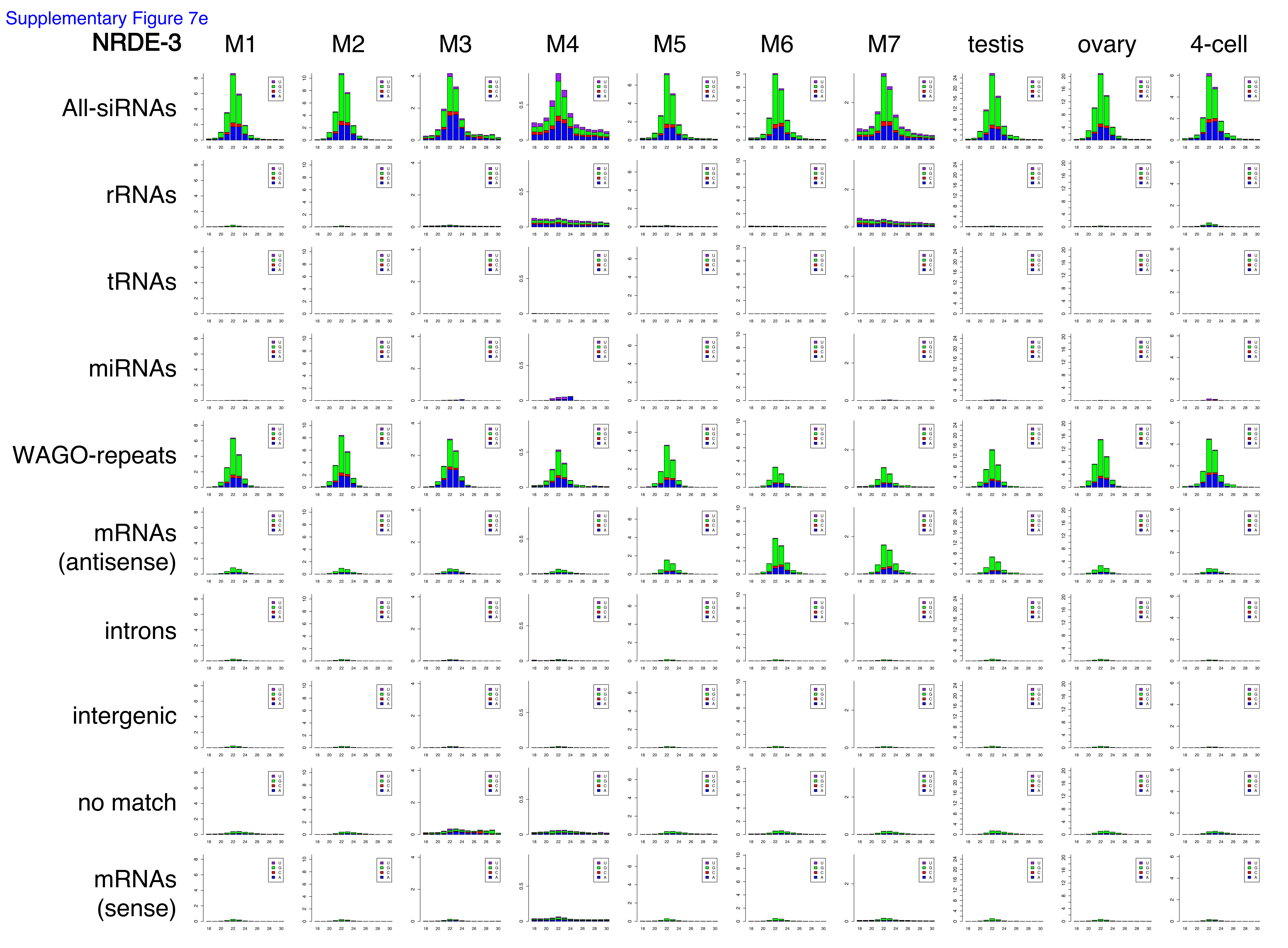
Supplementary Figure 7e
NRDE-3
M1
M2
M3
M4
M5
M6
M7
testis
ovary
4-cell
All-siRNAs
rRNAs
tRNAs
miRNAs
WAGO-repeats
mRNAs (antisense)
introns
intergenic
no match
mRNAs (sense)
U
G
C
A

Supplementary Figure 7f

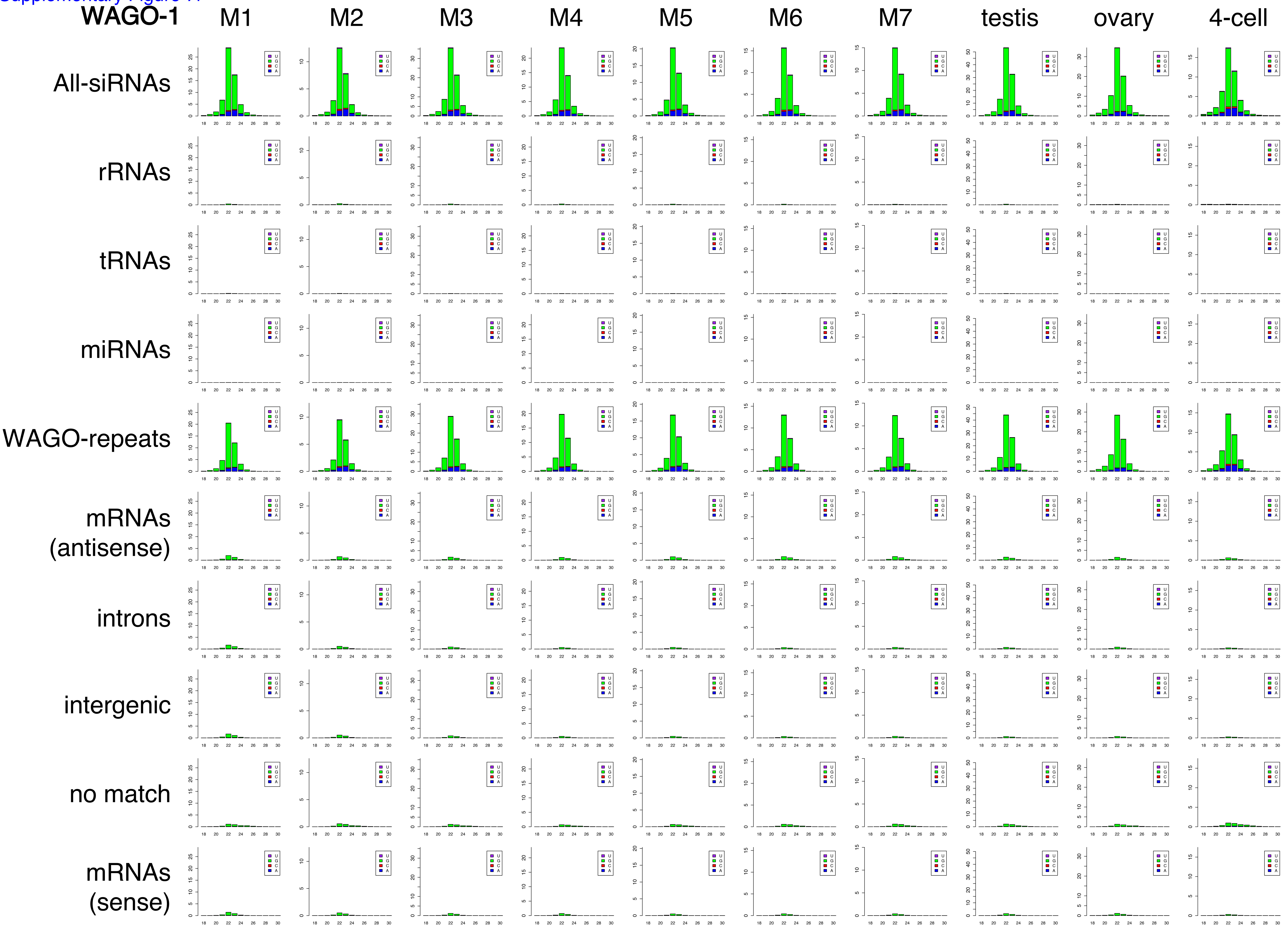

Supplementary Figure 7g

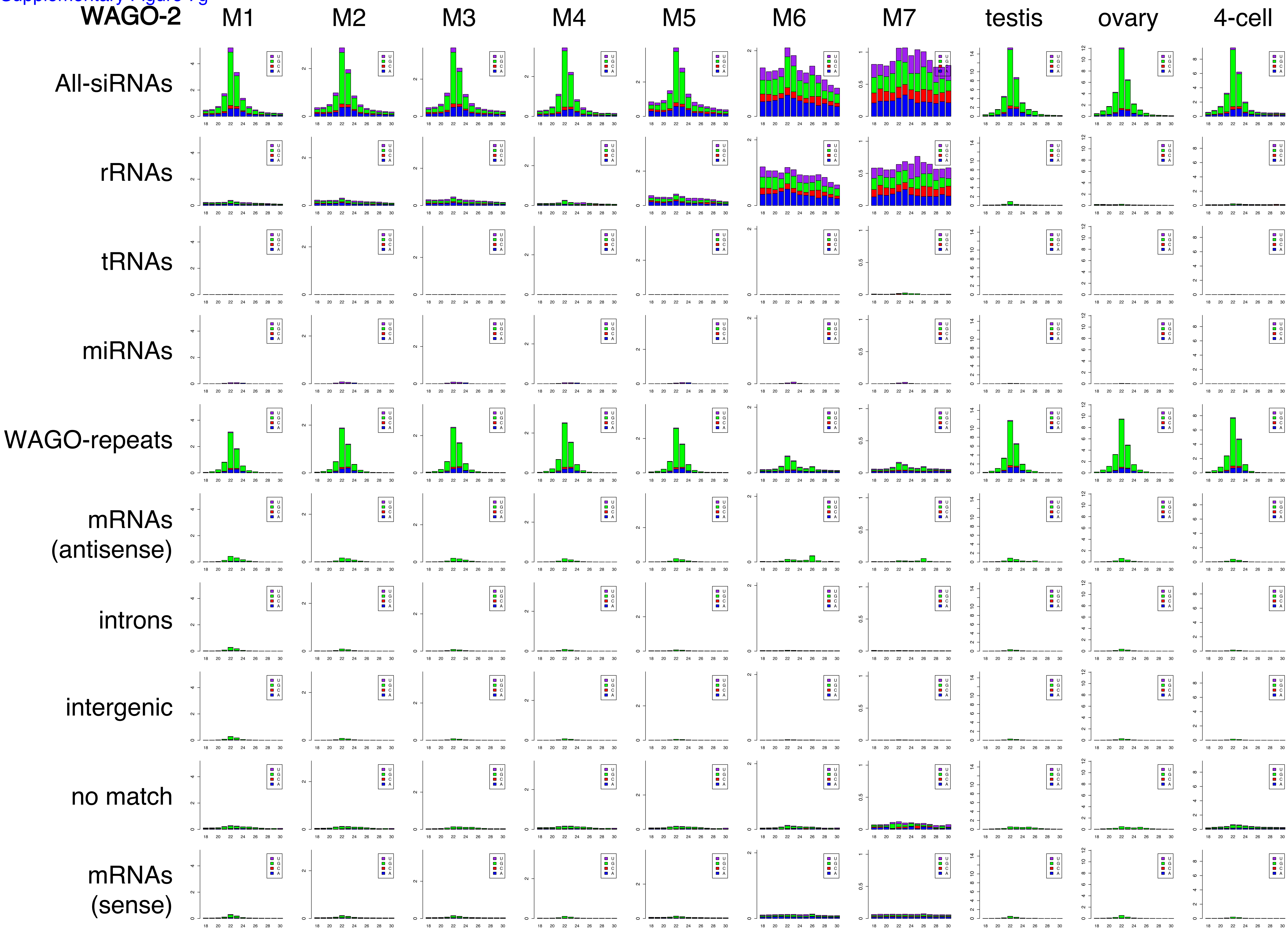

Supplementary Figure 8

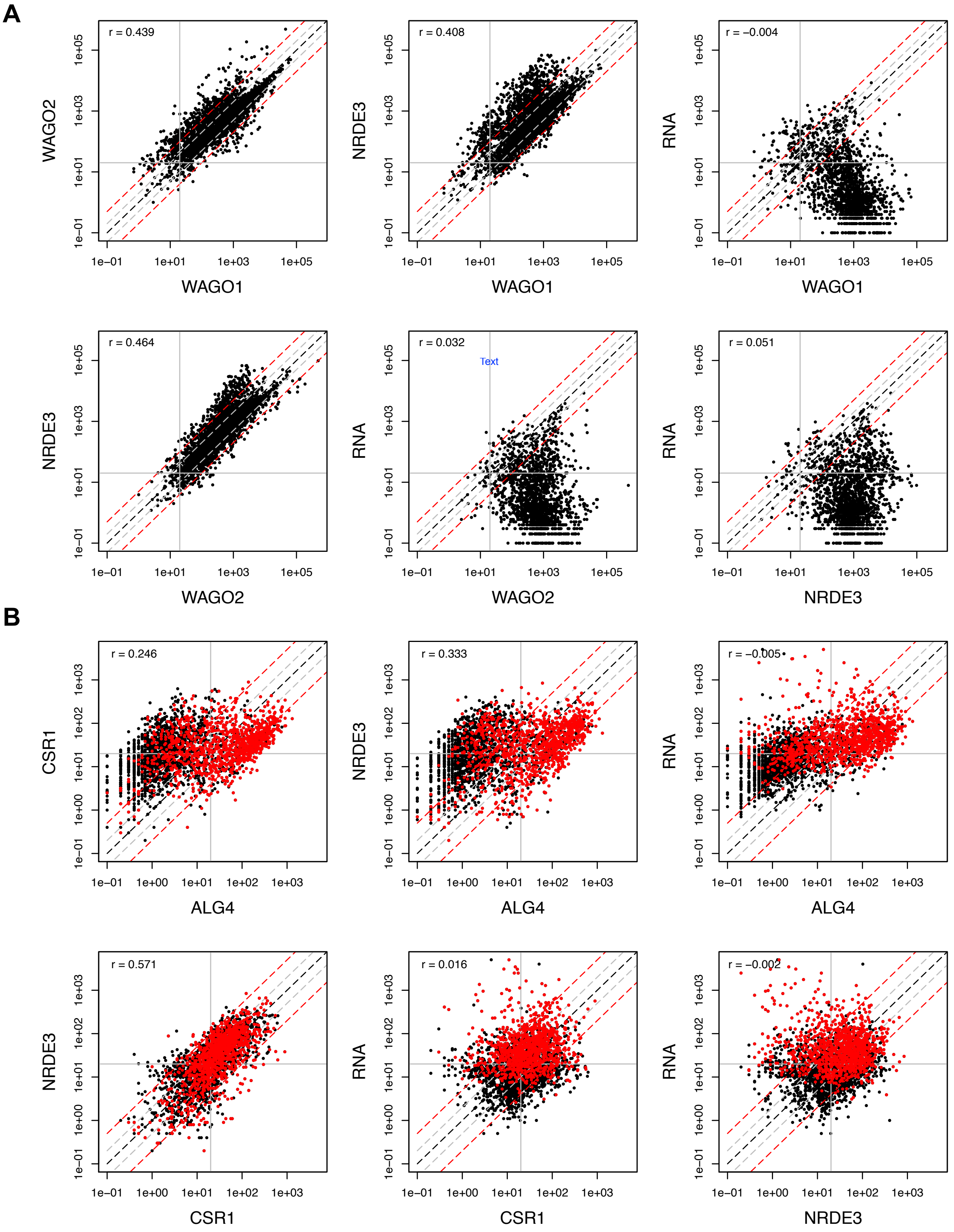

Supplementary Figure 9

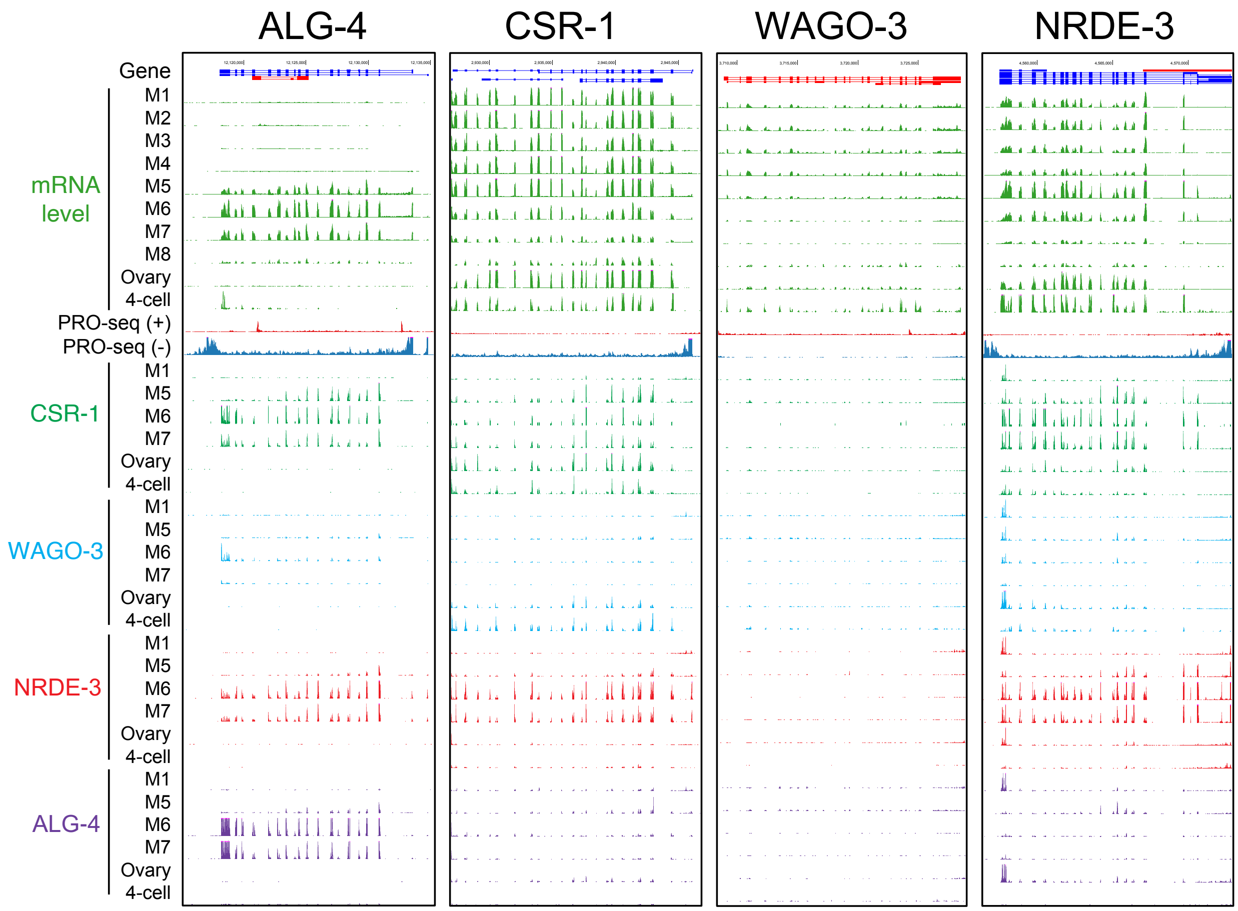

Supplement: Supplementary file 1 — Suppementary Information [file 41467_2022_28482_MOESM1_ESM.pdf]
