# Supplementary material for: Small RNA pathways in the nematode Ascaris in the absence of piRNAs
Source: Nat Commun. 2022 Feb 11;13:837. doi: 10.1038/s41467-022-28482-7 (PMC8837657; doi:10.1038/s41467-022-28482-7)
Supplement: Supplementary file 3 — Reporting Summary [file 41467_2022_28482_MOESM3_ESM.pdf]

nature portfolio

Corresponding author(s): Jianbin Wang and Richard E. Davis

Last updated by author(s): Dec 15, 2021

Reporting Summary

Nature Portfolio wishes to improve the reproducibility of the work that we publish. This form provides structure for consistency and transparency in reporting. For further information on Nature Portfolio policies, see our Editorial Policies and the Editorial Policy Checklist.

Statistics

For all statistical analyses, confirm that the following items are present in the figure legend, table legend, main text, or Methods section.

n/a	Confirmed	
☒	☐	The exact sample size (*n*) for each experimental group/condition, given as a discrete number and unit of measurement
☒	☐	A statement on whether measurements were taken from distinct samples or whether the same sample was measured repeatedly
☒	☐	The statistical test(s) used AND whether they are one- or two-sided<br>*Only common tests should be described solely by name; describe more complex techniques in the Methods section.*
☒	☐	A description of all covariates tested
☒	☐	A description of any assumptions or corrections, such as tests of normality and adjustment for multiple comparisons
☒	☐	A full description of the statistical parameters including central tendency (e.g. means) or other basic estimates (e.g. regression coefficient) AND variation (e.g. standard deviation) or associated estimates of uncertainty (e.g. confidence intervals)
☒	☐	For null hypothesis testing, the test statistic (e.g. *F*, *t*, *r*) with confidence intervals, effect sizes, degrees of freedom and *P* value noted<br>*Give P values as exact values whenever suitable.*
☒	☐	For Bayesian analysis, information on the choice of priors and Markov chain Monte Carlo settings
☒	☐	For hierarchical and complex designs, identification of the appropriate level for tests and full reporting of outcomes
☒	☐	Estimates of effect sizes (e.g. Cohen's *d*, Pearson's *r*), indicating how they were calculated

Our web collection on statistics for biologists contains articles on many of the points above.

Software and code

Policy information about availability of computer code

Data collection	no software was used for data collection
Data analysis	bowtie v1.2.2; bowtie2 v2.3.2; and bedtools v2.26.0-129-gc8b58bc for sequence analyses; TreeView for heatmaps; Clustal Omega, ClustalW , ClustalX, Muscle Multiple Sequence Alignment; Maximum Likelihood Phylogeny; MAFFT; PRANK Maximum Likelihood; NG Phylogeny FASTME, PhyML, PhyML+SMS, and FASTME; and iTOL for Phylogenetic analyses and trees; Applied Precision's Softworx software, Fiji software, and LASX software for image analysis.

For manuscripts utilizing custom algorithms or software that are central to the research but not yet described in published literature, software must be made available to editors and reviewers. We strongly encourage code deposition in a community repository (e.g. GitHub). See the Nature Portfolio guidelines for submitting code & software for further information.

Data

Policy information about availability of data

All manuscripts must include a data availability statement. This statement should provide the following information, where applicable:

- Accession codes, unique identifiers, or web links for publicly available datasets
- A description of any restrictions on data availability
- For clinical datasets or third party data, please ensure that the statement adheres to our policy

The small RNA and RNA sequencing data are deposited to the NCBI GEO database Accession GSE189061 and accessible at https://www.ncbi.nlm.nih.gov/geo/query/acc.cgi?acc=GSE189061. The data are also available in UCSC Genome Browser track data hubs that can be accessed with this link: http://genome.ucsc.edu/s/jianbinwang/Ascaris_small_RNAs.

Field-specific reporting

Please select the one below that is the best fit for your research. If you are not sure, read the appropriate sections before making your selection.

☒ Life sciences ☐ Behavioural & social sciences ☐ Ecological, evolutionary & environmental sciences

For a reference copy of the document with all sections, see nature.com/documents/nr-reporting-summary-flat.pdf

Life sciences study design

All studies must disclose on these points even when the disclosure is negative.

Sample size	Germline regions from 5 worms were pooled. Each male germline region is ~1-10 million cells and each female germline region >10 million cells. 15 million embryos were used per immunoprecipitation experiment.
Data exclusions	None
Replication	Minimum of two replicates with all replicates successful
Randomization	Random
Blinding	Blinded

Reporting for specific materials, systems and methods

We require information from authors about some types of materials, experimental systems and methods used in many studies. Here, indicate whether each material, system or method listed is relevant to your study. If you are not sure if a list item applies to your research, read the appropriate section before selecting a response.

Materials & experimental systems

n/a	Involved in the study	
☐	☒	Antibodies
☒	☐	Eukaryotic cell lines
☒	☐	Palaeontology and archaeology
☐	☒	Animals and other organisms
☒	☐	Human research participants
☒	☐	Clinical data
☒	☐	Dual use research of concern

Methods

n/a	Involved in the study	
☒	☐	ChIP-seq
☒	☐	Flow cytometry
☒	☐	MRI-based neuroimaging

Antibodies

Antibodies used	Antibodies used were all generated by the authors using proteins or peptides and commercial antibody services for the animals.
Validation	All antibodies were validated using several approaches (Western Blots, IP and MS, IHC, and IP and small RNA sequencing) and these data are described and presented in the manuscript itself. Antibody dilutions for IHC (1:100 - 1:500) or amounts for IP (5-10 µg) are described in the Methods.

Animals and other organisms

Policy information about studies involving animals; ARRIVE guidelines recommended for reporting animal research

Laboratory animals	None
Wild animals	Ascaris were collected from the intestines of pigs at slaughterhouses
Field-collected samples	Ascaris worms were recovered from the intestines of pigs into 37 C 1x PBS in insulated containers, transported warm to the laboratory, and germline and other samples dissected from the worms within 6 hours. Eggs were collected from fresh female worms or worms previously collected and kept cold for several days.
Ethics oversight	No ethical approval or guidance was required. The study did not include any sensitive objects or subjects.

Note that full information on the approval of the study protocol must also be provided in the manuscript.
